# Aphid Odorant-Binding Protein 9 Is Narrowly Tuned to Linear Alcohols and Aldehydes of Sixteen Carbon Atoms

**DOI:** 10.3390/insects12080741

**Published:** 2021-08-18

**Authors:** Chiara D’Onofrio, Wolfgang Knoll, Paolo Pelosi

**Affiliations:** 1 Biosensor Technologies, AIT Austrian Institute of Technology GmbH, Konrad-Lorenz Straße 24, 3430 Tulln, Austria; chiara.donofrio@ait.ac.at (C.D.); wolfgang.knoll@ait.ac.at (W.K.); 2 Department of Physics and Chemistry of Materials, Faculty of Medicine/Dental Medicine, Danube Private University, 3500 Krems, Austria

**Keywords:** odorant-binding protein, aphids, 1-hexadecanol, lepidopteran sex pheromones, ligand-binding assays, site-directed mutagenesis

## Abstract

**Simple Summary:**

Odorant-binding proteins (OBPs) mediate chemical communication in insects and can provide a shortcut to deciphering their olfactory code. Although being less specific than olfactory receptors, they are easy to express, purify and characterize. Aphids, in particular, are endowed with a limited repertoire of only 10 OBPs, which are exceptionally well conserved across species. Therefore, using OBPs to study olfaction in aphids appears to be a very attractive strategy. Within our project to functionally characterize all OBPs of the pea aphid, *Acyrthosiphon pisum*, we have expressed OBP9 and found it to be narrowly tuned to the linear alcohols and aldehydes of 16 carbon atoms. 1-Hexadecanol has been reported as a strong feeding repellant produced by toxic fungi. On the other hand, 16-carbon linear aldehydes are components of several lepidopteran pheromones and could represent warning signals to avoid potential feeding competition.

**Abstract:**

Aphid odorant-binding protein 9 is almost exclusively expressed in antennae and is well conserved between different aphid species. In order to investigate its function, we have expressed this protein and measured ligand-binding affinities to a number of common natural compounds. The best ligands are long-chain aldehydes and alcohols, in particular Z9-hexadecenal and Z11-hexadecenal, as well as 1-hexadecanol and Z11-1-hexadecenol. A model of this protein indicated Lys37 as the residue that is likely to establish strong interactions with the ligands, probably a Schiff base with aldehydes and a hydrogen bond with alcohols. Indeed, when we replaced this lysine with a leucine, the mutated protein lost its affinity to both long aldehydes and alcohols, while the binding of other volatiles was unaffected. Long-chain linear alcohols are common products of molds and have been reported as aphid antifeedants. Corresponding aldehydes, instead, are major components of sex pheromones for several species of Lepidoptera. We speculate that aphids might use OBP9 to avoid mold-contaminated plants as well as competition with lepidopteran larvae.

## 1. Introduction

Aphids are worldwide pests and are represented by around 5000 species feeding on most cultivated plants [1,2]. As well as the damage made to plants by direct feeding, aphids are carriers of serious pathogens. Given the wide variety of aphid species and of their host plants, the only efficient approach to control their populations is the use of insecticides, which are toxic to humans and other animals and eventually induce resistance [3,4].

Environmentally friendly alternatives are currently being investigated and include the action of natural enemies or of repellents. Coccinellidae and hoverflies are aphid predators, and strategies to attract these natural enemies to protect plants are promising, although so far they have not yet found effective practical applications. Parasitoids also represent an alternative to reduce aphid populations on cultivated plants [5,6,7,8].

The use of repellents, such as (*E*)-β-farnesene (EBF)—the alarm pheromone for most aphid species [9,10,11,12]—also proved ineffective. Based on the observation that some plants, such as the wild potato, protect themselves from aphid attacks by making and releasing EBF [13], transgenic plants have been prepared by adding enzymatic machinery for the synthesis of EBF [14,15]. However, the strategy was not successful in keeping aphids away, probably because aphids are able to distinguish the source of EBF on the basis of the timing of these chemical signals. In fact, in the presence of danger, aphids emit pulses of EBF to alert their conspecifics, while in transgenic plants, EBF is synthesized and released continuously [16]. These observations reveal how complex the recognition of chemical stimuli can be, even in simple insects such as aphids, and prompt us to study in detail their olfactory system at the molecular level.

To monitor chemical signals, such as environmental odors and pheromones, aphids, like all insects, use membrane-bound olfactory receptors (ORs) that are responsible for the fine recognition of chemical messengers, as well as soluble binding proteins that are involved in solubilizing and ferrying hydrophobic volatile molecules to the dendrites of olfactory neurons [17]. These soluble proteins are much easier to express and study than ORs and represent easier targets for understanding and interfering with the insect’s chemical language.

The pea aphid is equipped with 77 genes encoding gustatory receptors (GRs) and 79 encoding odorant receptors (ORs) [17,18]. By contrast, the repertoire of soluble carrier proteins is quite limited. Only 10 genes encode OBPs, apart from five isoforms of OBP3 and two additional members of uncertain classification [19]. OBPs of aphids are highly conserved among species, but their sequences are very different within the same species. Such a phenomenon is general with insect OBPs, but in aphids, it is particularly evident with identity values between members of the same clade that are often higher than 90% and sometime reaching 100%.

In addition, the repertoire of soluble carrier proteins also includes 10 chemosensory proteins (CSPs) [19] and two members of the Niemann–Pick C2 family [20]. Although some CSPs are likely involved in chemodetection, this protein family includes members playing different functions in insects, such as pheromone delivery, uptake of nutrients, insecticide resistance and development, among others [21,22].

The exceptional conservation of OBPs across aphids has been related to the fact that 16 species of aphids out of the 23 examined share the same alarm pheromone, (*E*)-β-farnesene (EBF), while in five, this volatile is present as a secondary component [1,2]. On the other hand, the sex pheromone blends of all aphid species are mixtures of different combinations and proportions of only four components, nepetalactone and three isomers of nepetalactol [23,24].

Therefore, the olfactory system of the aphid, due to its simplicity at the level of soluble carrier proteins, and in particular of OBPs, appears particularly attractive to decode the olfactory language used by these insects. In addition, the high similarity of sequences across species suggests similar binding characteristics and common physiological functions across aphid species. Defining the spectrum of ligands for each OBP will provide a key to the chemical code used by aphids to explore the chemical world and, consequently, might offer us the opportunity of interfering with their chemical communication system in order to control populations of these agricultural pests.

Thus far, OBP3 and OBP7 have been clearly shown to be linked to the detection of EBF. Such hypothesis was first based on ligand-binding experiments performed with six OBPs (OBP1, 3, 6, 7, 8 and 10) and a large number of EBF derivatives with repellent properties [25,26]. The involvement of OBP3 and OBP7 was further confirmed by experiments with aphids in which the expression of one or both genes encoding the two proteins had been inhibited. In particular, the typical escape reaction to EBF was lost only when both genes were silenced, thus indicating that OBP3 and OBP7 work as a backup of one another, and that other carrier proteins are not involved in such behavior [27].

In this work, we focus on the OBP9 of the pea aphid *Acyrthosiphon pisum*. A transcriptome analysis, performed on the English grain aphid *Sitobion avenae*, indicated that OBP9 is mainly expressed in the antennae of both winged and wingless individuals and, to lower levels, in legs as well [28]. Another work focused on the same species reported binding of the recombinant OBP9 to some plant volatiles, including lower linear alcohols and aldehydes for which poor-to-medium affinities were measured [29]. Very recently, the OBP9 of the peach aphid *Myzus persicae* was reported to bind EBF and hypothesized to work in cooperation with OBP3 and OBP7 [30].

Therefore, we decided to better explore the behavior of OBP9 in our model species, *A. pisum*, within a plan to map the specificities of all OBPs of the pea aphid. Ligand-binding experiments with the recombinant protein have revealed that OBP9 is tuned to linear aldehydes and alcohols of 16 carbon atoms, which in nature are components of sex pheromone blends for several species of Lepidoptera. In addition, 1-hexadecanol is a major volatile produced by molds and acts as antifeedant in different species of aphids [31,32]. By detecting these molecules, aphids might identify potentially toxic leaves, as well as the presence of lepidopteran larvae, which are likely competitors on the same host plant.

## 2. Materials and Methods

### 2.1. Chemicals

All chemicals were purchased from Merck KGaA, (Darmstadt, Germany), and were of analytical grade, except for methanol used to dilute odorants, which was of spectroscopic grade (Uvasol). Acrylamide and affinity columns for His-tag protein purification were from Bio-Rad, Vienna, Austria. Long-chain unsaturated aldehydes and alcohol were from Bedoukian (Danbury, CT, USA). Oligonucleotides were custom synthesized at Eurofins Genomics (Ebersberg, Germany). All enzymes and kits for DNA purification were from New England Biolabs (Ipswich, MA, USA).

### 2.2. RNA Extraction, cDNA Synthesis and Cloning

Total RNA was extracted from whole insects using the TRI reagent (Merck KGaA, Darmstadt, Germany) following the manufacturer’s procedure. The cDNA was synthesized using 2 µL of total RNA with the kit qScript cDNA SuperMix (Quanta Bio, Beverly, MA, USA), according to enclosed protocol. PCR was performed on cDNA using specific primers at the 5′ end and at the 3′ end (forward: 5′-AAGGATCCGATGATGCAGATGCAAAGGA-3′; reverse: 3′-AAGAATTCTTATTTTGATTTTGGTTTCAT-5′). The primers contained the restriction sites, BamHI and EcoRI, at the 5′ and the 3′ ends, respectively. The PCR product was digested with BamHI and EcoRI, purified and then ligated into a pET30 vector previously linearized with the same enzymes. After transformation of DH5α competent cells, plating and screening by PCR, positive colonies were grown, plasmids were extracted and custom sequenced at Eurofins Genomics (Ebersberg, Germany).

### 2.3. Preparation of Mutant

The plasmid pET30 containing the insert encoding ApisOBP9 was PCR amplified with the following primers: forward: 5′-ATGATAACAACTCTATATGACATA-3′, reverse: 3′-AAGAATTCTTATTTTGATTTTGGTTTCAT-5′ and using the following temperature program: 95 °C for 5 min; 35 cycles of 95 °C for 30 s, 50 °C for 30 s, 72 °C for 1 min; 72 °C for 10 min. Then, without purification, the product was submitted to a second PCR: 98 °C for 5 min; 20 cycles of 98 °C for 1 min, 72 °C for 7 min; 72 °C for 10 min. The crude product of the second PCR was digested with DpnI to degrade the original plasmid and used to transform DH5α *E. coli* competent cells. After plating, selected colonies were grown and sequenced. Those bearing the desired mutation were used to express the protein.

### 2.4. Protein Expression and Purification

After growing the culture to OD (600 nm) around 0.8, 0.4 mM IPTG was added to induce the expression, and the cells were further processed for 3 h at 37 °C. After centrifugation, the pellet was sonicated and centrifuged again. The recombinant proteins were found in the pellet and were solubilized in 8 M urea and 5 mM DTT for 1 h at room temperature, then they were dialyzed three times against 50 mM Tris-HCl buffer, pH 7.4. Purification of the solubilized proteins was accomplished by affinity chromatography on Bio-Scale^TM^ Mini Profinity^TM^ IMAC cartridge (1 mL, Bio-Rad Laboratories Ges.m.b.H., Vienna, Austria) according to the manufacturer’s instructions. The proteins were finally digested with Enterokinase (New England Biolabs) in order to remove the His-tag.

### 2.5. Ligand-Binding Assays

Affinity constants were evaluated by the competitive fluorescent method, using a PerkinElmer FL 6500 spectrofluorometer in a right-angle configuration at room temperature and quartz cuvettes with a 1 cm path. The fluorescent probe N-phenyl-1-naphthylamine (1-NPN) was excited at 337 nm, and emission spectra were recorded between 380 and 450 nm. Binding of 1-NPN was evaluated by adding aliquots of a 1 mM methanol solution to reach final concentrations of 2–16 μM, to a 2 μM solution of the protein in 50 mM Tris-HCl buffer, pH 7.4. Intensity values were recorded at the peak maximum, around 412 nm. The dissociation constant of the complex protein/1-NPN was calculated using Prism software (https://www.graphpad.com/scientific-software/prism/, accessed on 10 February 2021). The affinities of other ligands were evaluated in competitive binding assays by adding aliquots of 1 mM methanol solutions of each ligand to final concentration values of 2 to 16 μM, to a mixture of the protein and 1-NPN, both at the concentration of 2 μM in 50 mM Tris-HCl buffer, pH 7.4. Dissociation constants of ligands were calculated from the corresponding [IC]_50_ values (the concentration of each ligand halving the initial value of fluorescence), using the equation:

K_D_ = [IC]_50_/1 + [1-NPN]/K_1-NPN_

where [1-NPN] is the concentration of free 1-NPN, and K_1-NPN_ the dissociation constant of the complex protein/1-NPN.

### 2.6. Modelling and Docking

A three-dimensional model of ApisOBP9 was obtained with the on-line program SWISS MODEL [33,34,35] using, as a template, the pheromone-binding protein of the honey bee ASP1 (PDB: 3cdnA) [36]. Docking was simulated with the on-line program SWISS DOCK using default parameters [37]. Models were visualized with the UCSF Chimera package. Chimera is developed by the Resource for Biocomputing, Visualization, and Informatics at the University of California, San Francisco (supported by NIGMS P41-GM103311) [38].

## 3. Results

### 3.1. Expression and Purification

**Figure 1:**
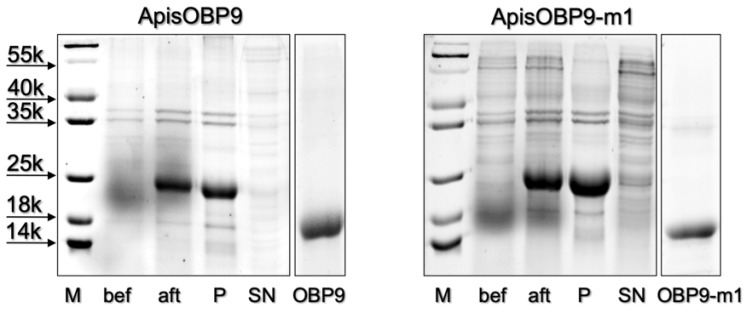
Expression and purification of ApisOBP9 and its mutant ApisOBP9-m1 (K37L). Samples before (bef) and after (aft) the induction with IPTG were centrifuged and the pellets dissolved in sample buffer and analyzed by SDS-PAGE. P and SN refer to samples of the pellet and the supernatant after sonication and centrifugation. The analysis of the protein samples obtained after removal of His-tag and final purification are shown in the last lanes.

The OBP9 of *A. pisum* (ApisOBP9) was prepared from RNA extracted from whole individuals, as detailed in the Section 2. The construct included a His-tag at the N-terminus, which was removed after purification of the protein. Figure 1 reports the electrophoretic separation of samples at different stages of the expression and purification process. The purified protein was then used in ligand-binding experiments.

### 3.2. Ligand-Binding Assays

**Figure 2:**
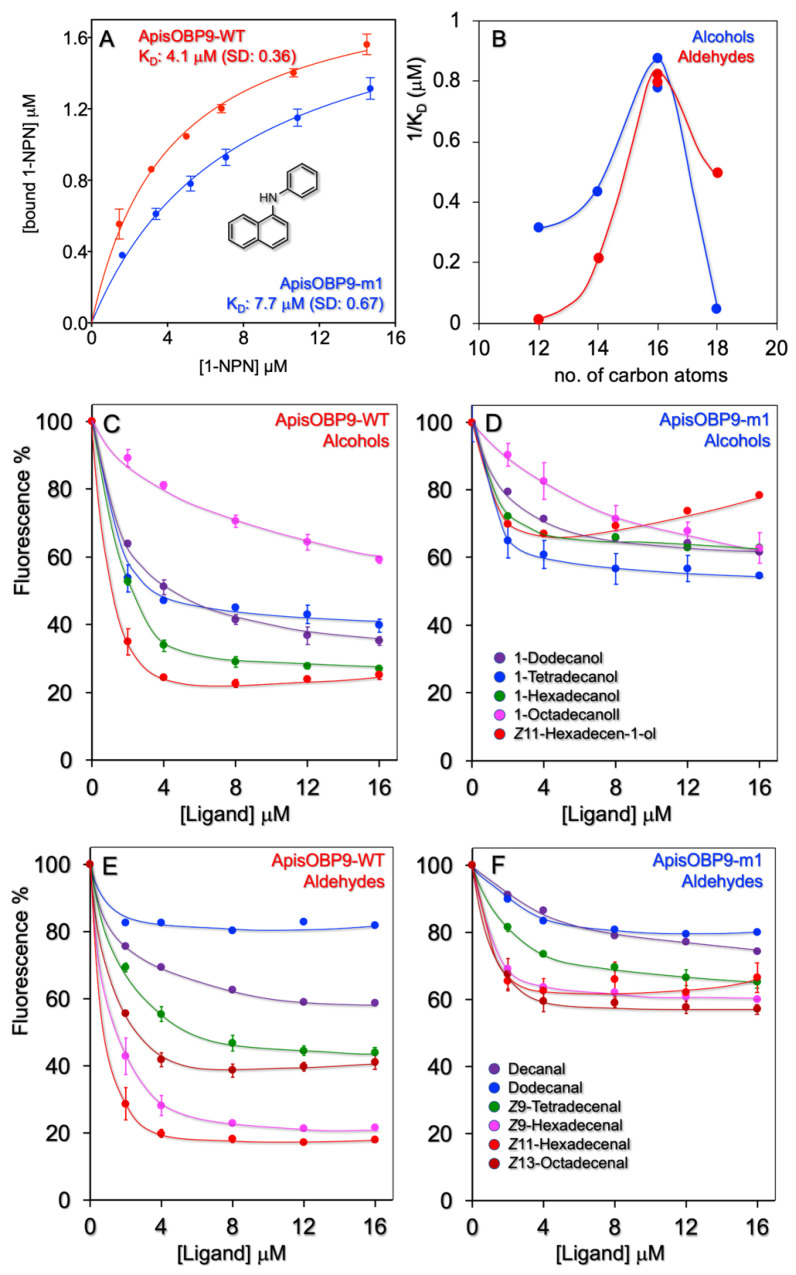
Ligand-binding experiments with ApisOBP9 and its mutant ApisOBP9-m1 (K37L). (**A**). Both WT and mutant ApisOBP9 bind 1-NPN with good affinities. (**C**,**E**). ApisOBP9 binds linear alcohols and aldehydes of 14–18 carbon atoms. (**B**). The protein is narrowly tuned to 16-carbon linear alcohols and aldehydes. (**D**,**F**). Replacing Lys37 with Leu in ApisOBP9-m1 had the effect of strongly reducing the affinity to long-chain alcohols and aldehydes, specifically to the 16-carbon members.

All results of the binding experiments are reported in Figure 2 and in Supplementary Figures S1–S4, including those performed on a mutant (Lys37Leu), designed on the results obtained with the wild-type ApisOBP9. Supplementary Table S1 provides a list of all the ligands used in the binding experiments.

First, we measured the affinity of the protein to the fluorescent probe N-phenyl-1-naphthylamine (1-NPN), the most widely used with insect OBPs. ApisOBP9 binds 1-NPN with a dissociation constant of 4.1 μM, thus enabling the use of competitive binding experiments to estimate the affinity of other ligands (Figure 2A).

We have tested a number of pure volatiles belonging to different chemical classes, including the terpenoids and aromatics that are commonly found among plant volatiles (Supplementary Figures S1 and S2, and Table S1). None of these compounds proved to be a good ligand for ApisOBP9, with only coniferyl aldehyde and safranal showing moderate affinities. Then, we tested series of linear alcohols, aldehydes, esters and carboxylic acids (Figure 2, Supplementary Figures S3 and S4 and Table S1). We measured good affinities between ApisOBP9 and long-chain alcohols and aldehydes, with the best ligands being the members with 16 carbon atoms, as graphically illustrated in the plot of Figure 2B. The relative displacement curves are reported in Figure 2C,E. Long-chain esters proved to be weak ligands (Supplementary Figure S3). Carboxylic acids of the same lengths, on the other hand, did not show significant affinity to ApisOBP9.

Therefore, ApisOBP9 appears to be rather narrowly tuned to linear alcohols and aldehydes of 16 carbon atoms. In fact, the addition or deletion of two carbons from the chain is enough to strongly reduce the affinity to the protein.

**Figure 3:**
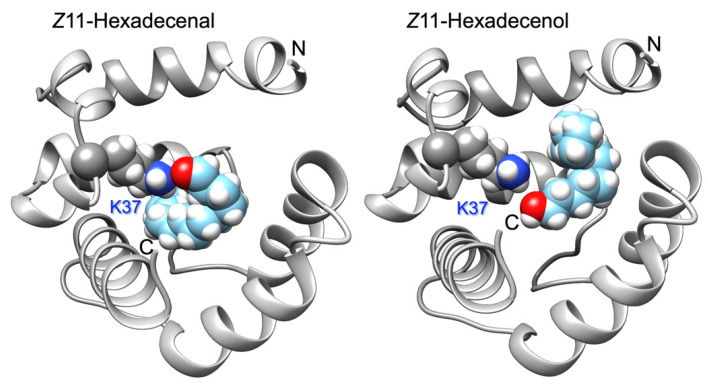
Docking of the best ligands, Z11-hexadecenal and Z11-hexadecenol, to a model of ApisOBP9. Both ligands fit inside the binding cavity with their functional groups at short distance from the ε-amino group of Lys37. N- and C-termini are indicated. The model was obtained using the on-line program Swiss-Model. Docking simulations were performed with the program Swiss-Dock. Structures were visualized using the software Chimera.

Based on a model built on the template structure of the pheromone-binding protein of the honey bee ASP1 (PDB: 3cdnA) [36], we hypothesized that Lys37 could be responsible for relatively strong interactions with the aldehyde carbonyl group possibly through formation of a Schiff base, or through a hydrogen bond with the hydroxyl group of alcohols. Docking simulations supported such view (Figure 3).

To verify our hypothesis, we prepared a mutant of ApisOBP9 by replacing Lys37 with Leu. The idea was to eliminate the charge and any group capable of hydrogen bonding, while keeping, to some extent, the steric characteristics of the residue. However, replacing a charged group chain with a hydrocarbon chain might upset the entire scaffolding of the protein. To verify this aspect, we used the software PoPMuSiC [39] to check for the stability of the mutated protein. This simulation predicts that the replacement of Lys37 with Leu would result in a slight destabilization (ΔΔG = 0.11) but would likely not strongly affect the scaffolding of ApisOBP9. On the other hand, as Lys37 lies at the mouth of the binding pocket, its replacement with a hydrophobic residue might facilitate the entrance of hydrophobic ligands [40]. Therefore, in cases where Lys37 was not directly involved in binding, we would measure strong affinities of hydrophobic ligands to the mutated protein. To verify such hypothesis, we prepared the K37L mutant, as described in the Section 2 and measured its affinity in competitive binding assays to a selection of ligands used with the WT protein.

ApisOBP9-K37L showed drastically reduced binding to long-chain aldehydes and alcohols (Figure 2, panels D and F)—as well as, to some extent, to esters (Supplementary Figure S3)—thus supporting our hypothesis. Instead, affinities to other ligands, including safranal and coniferyl aldehyde (Supplementary Figure S2), as well as shorter aldehydes and alcohols (Figure 2), were not significantly affected. Thus, although the replacement of a lysine with a leucine might facilitate the entrance of hydrophobic ligands, its negative effect on binding strongly indicates that Lys37 is involved in the formation of stable complexes with alcohols and aldehydes of 16 carbon atoms.

## 4. Discussion

In this work, we have measured the ligand-binding properties of ApisOBP9, one of the 10 OBPs of the pea aphid, and found that this protein is narrowly tuned to 16 carbon linear alcohols and aldehydes. Such specificity is supported by the observation that replacing Lys37 with Leu drastically reduces the affinity to those chemicals. The literature reports two studies performed with OBP9 of other aphid species. In the first, the orthologue protein of the English grain aphid *S. avenae* is reported to bind with poor-to-medium affinities, some lower linear alcohols and aldehydes [29]. In another paper, hexyl hexanoate and EBF were found to be the best ligands for OBP9 of the peach aphid *M. persicae* [30]. Neither of these two works tested long-chain alcohols and aldehydes; therefore, a comparison with our data is not feasible. However, in our hands, ApisOBP9 showed some affinity to EBF, although not as good as those observed with 16-carbon alcohols and aldehydes (Supplementary Figure S1). We suggest that such modest affinity of ApisOBP9 to EBF is rather accidental and is not linked to behavioral effects. In fact, silencing either ApisOBP3 or OBP7 reduces the avoidance behavior to EBF, while silencing both genes produces a complete loss of such behavior [27]. This not only indicates that both OBP3 and OBP7 are related to avoidance of EBF in the pea aphid, but it also excludes other proteins to mediate this behavior. Such a conclusion, however, cannot be extrapolated to other aphid species and does not disprove the hypothesis that, in the peach aphid, three OBPs might be involved in the detection of the alarm pheromone.

The fine tuning of ApisOBP9 to 16 carbon linear alcohols and aldehydes poses questions on the ecological meaning of these volatiles. Long-chain aldehydes are major components of sex pheromones for a large number of Lepidoptera. Detecting their pheromones might provide important cues to aphids either to avoid competition on the same leaf or else to recognize leaves that have already been attacked by caterpillars as good sites for feeding. On the other hand, 1-hexadecanol is a product of fungal infestation and has been reported to act as a deterrent for aphids. In a recent paper [32], several fungal metabolites, including 1-hexadecanol, were reported to act as strong repellents at concentrations around 1 mM for the pea aphid *A. pisum*. Another work performed with the bird cherry-oat aphid *Rhopalosiphum padi* [31] found that long-chain linear primary alcohols with 14–20 carbon atoms acted as feeding deterrents for both winged and wingless forms of this aphid at concentrations as low as 0.15 mM.

In conclusion, it seems reasonable to suggest that OBP9 mediates the avoidance of fungi-contaminated leaves by mediating the detection of 1-hexadecanol, one of the main fungi metabolites, and similar compounds. The same protein, which also shows high and specific affinity to 16-carbon linear aldehydes, could also mediate the avoidance of feeding sites that have already been colonized by lepidopteran larvae, but specific behavior experiments are needed before reaching any reasonable conclusion.

## Data Availability

All data can be found in the Supplementary Materials.

## Supplementary Materials

The following are available online at https://www.mdpi.com/article/10.3390/insects12080741/s1, Figure S1: Binding of common plant volatiles to ApisOBP9, Figure S2: Binding of safranal and coniferyl aldehyde to ApisOBP9 and ApisOBP9-m1, Figure S3: Binding of esters to ApisOBP9 and ApisOBP9-m1, Figure S4: Binding of fatty acids to ApisOBP9 and ApisOBP9-m1, Table S1: Ligands used in this study.
