# Supplementary material for: Aphid Odorant-Binding Protein 9 Is Narrowly Tuned to Linear Alcohols and Aldehydes of Sixteen Carbon Atoms
Source: Insects. 2021 Aug 18;12(8):741. doi: 10.3390/insects12080741 (PMC8396812; doi:10.3390/insects12080741)
Supplement: Supplementary file 1 [file insects-12-00741-s001.zip › insects-1336826-supplementary.pdf]

Aphid Odorant-Binding Protein 9 Is Narrowly Tuned to Linear Alcohols and Aldehydes of Sixteen Carbon Atoms

Chiara D'Onofrio[1], Wolfgang Knoll[1,2], Paolo Pelosi[1*]
[1]AIT Austrian Institute of Technology GmbH, Biosensor Technologies, Konrad-Lorenz Straße 24, 3430 Tulln, Austria.
[2]Department of Physics and Chemistry of Materials, Faculty of Medicine/Dental Medicine, Danube Private University, Krems, Austria
*Paolo Pelosi
Austrian Institute of Technology GmbH, Biosensor Technologies, Konrad-Lorenz Straße, 24, 3430 Tulln, Austria. E.mail: ppelosi.obp@gmail.com; paolo.pelosi@ait.ac.at. Tel. +39 347 060 4288

Supplementary Material

Supplementary Table S1. Ligands used in binding experiments and calculated dissociation constants with the WT and mutant (K37L) ApisOBP9. N.D.: not determined because at the highest concentration used quenching did not reach 50%. N.T.: not tested.

	Ligand	N. Carbons	ApisOBP9-WT	ApisOBP9-m1
Alcohols	1-Decanol	10	N.D.	N.D.
	1-Dodecanol	12	3.79	N.D.
	1-Tetradecanol	14	3.41	N.D.
	1-Hexadecanol	16	1.90	N.D.
	(*Z*)-11-Hexadecen-1-ol	16	1.37	N.D.
	1-Octadecanol	18	N.D.	N.D.
Aldehydes	Decanal	10	N.D.	N.D.
	Dodecanal	12	N.D.	N.D.
	(*Z*)-9-Tetradecenal	14	5.31	N.D.
	(*Z*)-9-Hexadecenal	16	1.44	N.D.
	(*Z*)-11-Hexadecenal	16	1.14	N.D.
	(*Z*)-13-Octadecenal	18	2.28	N.D.
Esters	Ethyl decanoate	12	N.D.	N.D.
	Ethyl laurate	14	N.D.	N.D.
	(*Z*)-7-Dodecenyl acetate	14	1.38	N.D.
	Ethyl myristate	16	N.D.	N.D.
	Ethyl palmitate	18	N.D.	N.D.
	(*Z*)-11-16-Acetate	16	N.D.	N.D.
Acids	Decanoic acid	10	14.5	N.D.
	Lauric acid	12	N.D.	N.D.
	Myristic acid	14	N.D.	N.D.
	Palmitic acid	16	N.D.	N.D.
	Stearic acid	18	N.D.	N.D.
	Oleic acid	18	N.D.	N.D.
	Linoleic acid	18	N.D.	N.D.
Other compounds	Coniferyl aldehyde	10	7.89	8.78
	Safranal	10	11.38	16.9
	Citral	10	N.D.	N.T.
	α-Methoxycinnamaldehyde	10	N.D.	N.T.
	Ocimene	10	N.D.	N.T.
	β-farnesene	15	N.D.	N.T.
	Farnesol	15	N.D.	N.T.
	Methyl jasmonate	13	N.D.	N.T.
	Methyl salicylate	8	N.D.	N.T.
	Neryl acetate	12	N.D.	N.T.
	Geranyl acetone	13	N.D.	N.T.
	Eugenol	10	N.D.	N.T.
	β-Ionone	13	N.D.	N.T.

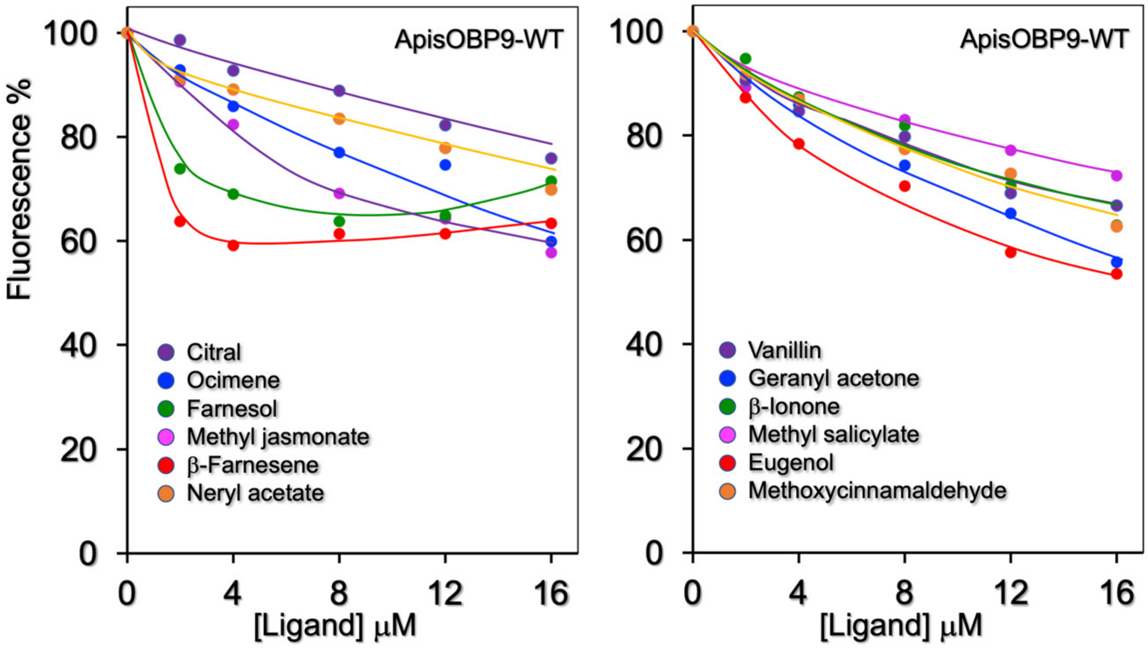

Supplementary Fig. S1. Competitive binding curves of common plant volatiles to ApisOBP9.

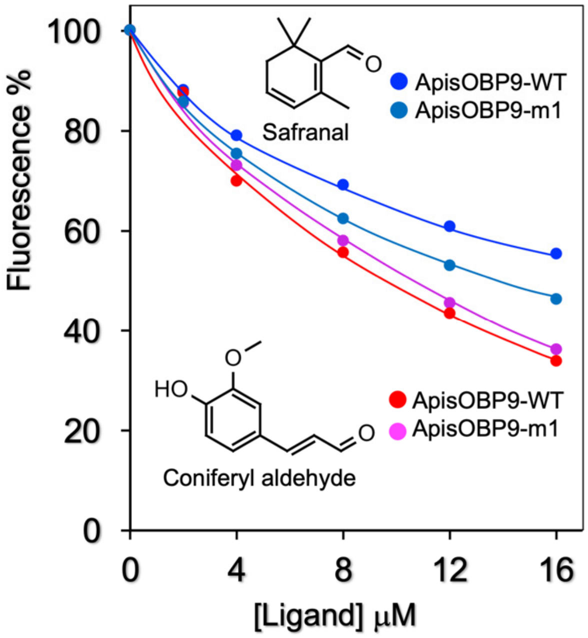

Supplementary Fig. S2. Competitive binding curves of safranal and coniferyl adehyde to ApisOBP9-WT and mutant ApisOBP9-m1 (K37L).

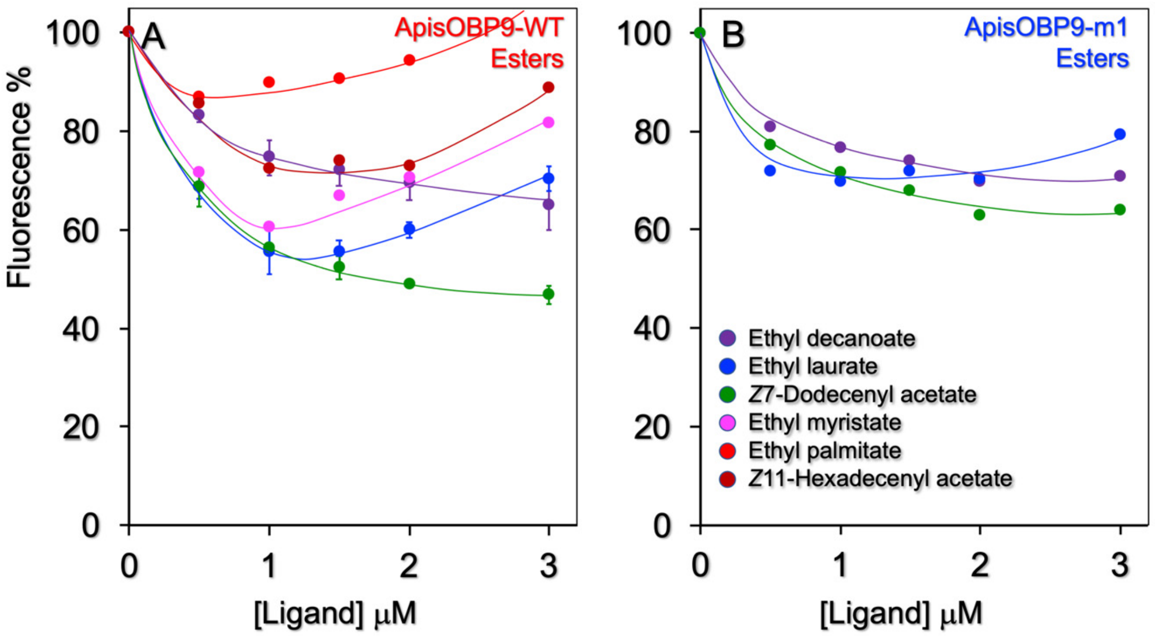

Supplementary Fig. S3. Competitive binding curves of some long-chain esters to ApisOBP9-WT and mutant ApisOBP9-m1 (K37L).

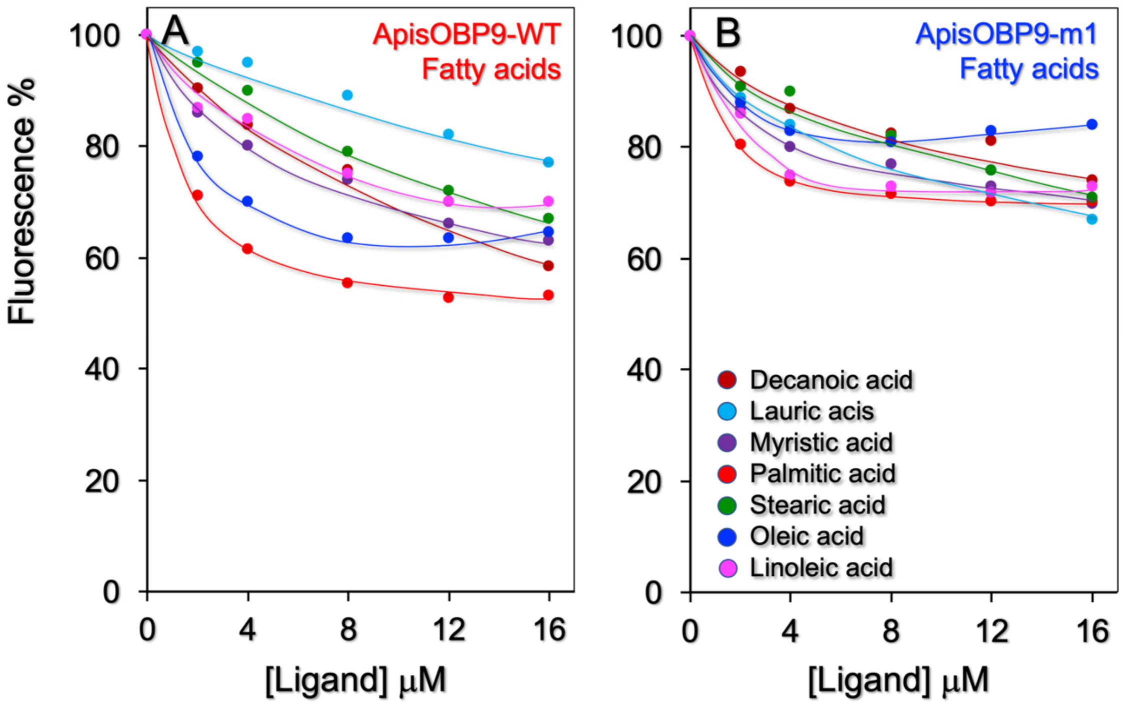

Supplementary Fig. S4. Competitive binding curves of some long-chain fatty acids to ApisOBP9-WT and mutant ApisOBP9-m1 (K37L).
